# Synaptic deficits in iPSC-derived cortical interneurons in schizophrenia are mediated by NLGN2 and rescued by *N*-acetylcysteine

**DOI:** 10.1038/s41398-019-0660-x

**Published:** 2019-11-28

**Authors:** Annie Kathuria, Kara Lopez-Lengowski, Bradley Watmuff, Donna McPhie, Bruce M. Cohen, Rakesh Karmacharya

**Affiliations:** 1 Center for Genomic Medicine, Massachusetts General Hospital, Boston, MA USA; 2 Chemical Biology and Therapeutic Science Program, Broad Institute of MIT & Harvard, Cambridge, MA USA; 3 Department of Psychiatry, Harvard Medical School, Boston, MA USA; 4 Schizophrenia and Bipolar Disorder Program, McLean Hospital, Belmont, MA USA; 5 Graduate Program in Chemical Biology, Harvard University, Cambridge, MA USA; 6 Program in Neuroscience, Harvard University, Cambridge, MA USA

**Keywords:** Molecular neuroscience, Schizophrenia

## Abstract

Human postmortem studies suggest a major role for abnormalities in GABAergic interneurons in the prefrontal cortex in schizophrenia. Cortical interneurons differentiated from induced pluripotent stem cells (iPSCs) of schizophrenia subjects showed significantly lower levels of glutamate decarboxylase 67 (GAD67), replicating findings from multiple postmortem studies, as well as reduced levels of synaptic proteins gehpyrin and NLGN2. Co-cultures of the interneurons with excitatory cortical pyramidal neurons from schizophrenia iPSCs showed reduced synaptic puncta density and lower action potential frequency. NLGN2 overexpression in schizophrenia neurons rescued synaptic puncta deficits while NLGN2 knockdown in healthy neurons resulted in reduced synaptic puncta density. Schizophrenia interneurons also had significantly smaller nuclear area, suggesting an innate oxidative stressed state. The antioxidant *N*-acetylcysteine increased the nuclear area in schizophrenia interneurons, increased NLGN2 expression and rescued synaptic deficits. These results implicate specific deficiencies in the synaptic machinery in cortical interneurons as critical regulators of synaptic connections in schizophrenia and point to a nexus between oxidative stress and NLGN2 expression in mediating synaptic deficits in schizophrenia.

## Introduction

Schizophrenia (SCZ) is a chronic and debilitating psychiatric disorder characterized by hallucinations, paranoid delusions, disordered thought processes, and cognitive deficits^1^. The onset of psychosis is typically in adolescence or early adulthood and it follows a chronic course requiring treatment for the rest of a person’s life^2,3^. Patients have an elevated risk of suicide compared to the general population, and suicide is the cause of over 10% of deaths in patients with psychotic disorders^4^. SCZ is a significant contributor to the global burden of disease—SCZ is the 8th leading cause of disability-adjusted life year worldwide and psychosis is ranked as the 3^rd^ most disabling condition^5,6^. The diagnosis and treatment of SCZ is based only on clinical symptomatology, current treatments are only partly effective, and there are no biomarkers to aid in diagnosis, in guiding treatment decisions or in monitoring treatment response. Despite the high prevalence and enormous impact, the disease biology of SCZ remains elusive^7^. There is an urgent need for understanding the cellular-molecular underpinnings of SCZ that can be leveraged for the development of novel therapeutics that can bring about meaningful improvement in the functional outcomes for patients with SCZ^8,9^.

Postmortem studies and animal models indicate that the balance of excitatory and inhibitory (E-I) activity of cortical circuits is altered in SCZ^10–12^. One of the most replicated postmortem findings in SCZ brains is evidence of GABAergic deficits in the prefrontal cortex that suggest a decrease in the activity of cortical interneurons^13–15^. Optogenetic studies in animals show that elevated excitation, but not elevated inhibition, in the prefrontal cortex lead to impaired cognition and social behaviour^16^. Deficits in GABAergic transmission tip the E-I balance in the cortex in this direction. In this study, we sought to develop ex vivo models of cortical interneuron cultures from human subjects in order to identify cellular and molecular substrates of SCZ disease biology. To that end, we have generated iPSCs from 9 subjects each with SCZ and healthy controls (CON) and differentiated them into cortical interneurons in order to examine disease-specific differences in the biology of inhibitory neurons.

## Materials and methods

### Differentiation of cortical interneurons from human iPSCs

iPSCs were cultured to 100% confluency and media changed to N2/B27, along with addition of 10 μM SB431542 (Sigma S4317), 2 μM XAV939 (Sigma X3004) and 1 μM dorsomorphin (Sigma P5499). Media was changed daily for 7 days and cells were split 1:1 onto Geltrex substrate on day 8. These neural progenitor cells were cultured in N2/B27 and split once cells were confluent. 1.5 μM purmorphamine (Sigma SML0868) was added during day 10–20, cells transferred on day 21 to plates coated with 10 μg/ml poly-L-ornithine (Sigma P3655) and 10 μg/ml laminin (Sigma L2020) and then cultured in BrainPhys media containing 10 μM DAPT (Sigma D5942).

### Immunocytochemistry

Cells were fixed with 4% paraformaldehyde at room temperature, washed with PBS, permeabilized in PBST (PBS + 0.1% Triton X) and blocked with PBS plus 5% goat serum. Fixed cells were incubated with primary antibodies plus 1% goat serum overnight at 4 °C, followed by PBS washes, and incubation with secondary antibodies plus 1% goat serum for 1 h at room temperature. Antibodies used are listed in Supplementary Table 1.

### Western blots

Samples were lysed and protein concentration measured with a BSA assay. In all, 10 μg of protein extract was run on each lane on a Criterion TGX Precast gel 4–20% (BIO-RAD 5671094). Gels were transferred to a Immobilon-P Transfer Membrane (Millipore IPVH00010, pore size: 0.45 μm, PVDF), membrane blocked in Odyssey Blocking Buffer (Li-Cor 927-40,000) and probed overnight with primary antibodies at 4 °C. Following washes with 1x TBST, membrane was incubated with secondary antibody using donkey anti-rabbit (Li-Cor 1:10,000, IRDye 680, 925-68073, lot #C70601-01) or anti-mouse (Li-Cor 1:10,000, IRDye 800, 925-32212, lot #C70502-03). Images from Li-Cor Odyssey Clx Imaging System were analysed and quantified using Image Studio Version 5.2. Antibodies are listed in Supplementary Table 1.

### Oxidative stress experiments

We used the ROS-ID® Total ROS/Superoxide detection kit (Enzo life ENZ-51010), which includes two fluorescent dyes—total reactive oxygen species (ROS) detection reagent (Green) and Superoxide Detection Reagent (Orange). Neurons were imaged using Opera Phenix high-content imaging system (Perkin Elmer).

### Image analysis

Dissociated neurons were plated on poly-L-ornithine and laminin-coated 24- or 96-well tissue culture plates. Cells were fixed and stained with neuronal marker MAP2. Quantitative image analyses of cortical interneuron cultures were conducted in Opera Phenix at 20x magnification using Harmony software (Perkin Elmer). Cell soma area, nuclear area and neurite length from 10 randomly selected fields were quantified.

### Quantification of synaptic puncta

Dissociated neurons were plated on poly-l-ornithine and laminin-coated glass bottom 24-well tissue culture plates. Cells were fixed and stained with neuronal markers MAP2, gephyrin, synaptotagmin1/2 and Homer 1. Image analyses were conducted in Opera Phenix at 60x magnification using Harmony software. Neurites and synaptic puncta were identified in an automated way based on synaptic marker staining along neurite length to calculate puncta density.

### qPCR

RNA was extracted using the RNeasy Mini Kit (Qiagen 74104) and 1 μg RNA was converted into cDNA using High Capacity cDNA Reverse Transcription Kit (ThermoFisher 4368814). SYBR green (Mangobio 08-25-00020) assay was run on Roche light cycler 480II. We used 96-well plates with each well containing 4 μl of EvaGreen master mix, 2 μl primer (2 μM), 5 μlDNA (16 ng/μl) and 9 μl water. Three technical replicates per sample were tested. Analysis was performed on Excel and graphs prepared with GraphPad version 8.0. Primers are listed in Supplementary Table 2.

### NLGN2 knockdown

Knockdown NLGN2 GFP shRNA lentiviral particles (Origene TL302944V) or scrambled GFP lentiviral particles were transduced in a co-culture of excitatory and inhibitory neurons from a control iPSC line at day 85 of differentiation. Knockdown was considered successful when NLGN2 antibody staining did not co-localize with GFP-positive cells. GFP-positive cells were analysed for synaptic puncta quantification.

### NLGN2 overexpression

Overexpression NLGN2 GFP lentiviral particles (Origene RC222544L2V) or scrambled GFP lentiviral particles were transduced in a co-culture of excitatory and inhibitory neurons from a SCZ iPSC line at day 85 of differentiation. Overexpression was considered successful when NLGN2 antibody staining co-localized with GFP-positive cells.

### Microelectrode array experiments

Microelectrode array (MEA) experiments were performed using a MED64 Presto. Co-cultured neurons at day 90 were plated on MEA 24-well plates, which has 16 electrodes per well. Spontaneous activity was recorded for 1-min periods and data analysed using MEA symphony software.

### Calcium imaging and analysis

We imaged interneurons at 37 °C in 1 μM Fluo-4AM solution for 60 min. The glass dish with the cells was mounted on stage of the Leica TCS SP8. Exposures (ex/em 494/506 nm) were captured every two seconds for 90 s before and after addition of 30 mM GABA. To record responses from specific neurons, the experiment was “replayed” using ImageJ and regions of interest (ROIs) drawn around neurons to measure fluorescence intensity. Collected data was used to calculate magnitude of fluorescence change by dividing fluorescence intensity after addition of GABA (F) with baseline fluorescence intensity (F0).

### Data collection and statistics

All experiments were repeated at least three times. Only neuronal cultures with >80% MAP2 positive cells were used. Cultures were randomly selected for assays in a blinded manner. Statistical analyses used are reported in figure legends. Normal distribution was checked using the Kolmogorov-Smirnov’s test. Statistical analyses were done with Prism8.

## Results

### Generation of cortical interneurons from iPSCs

**Fig. 1:**
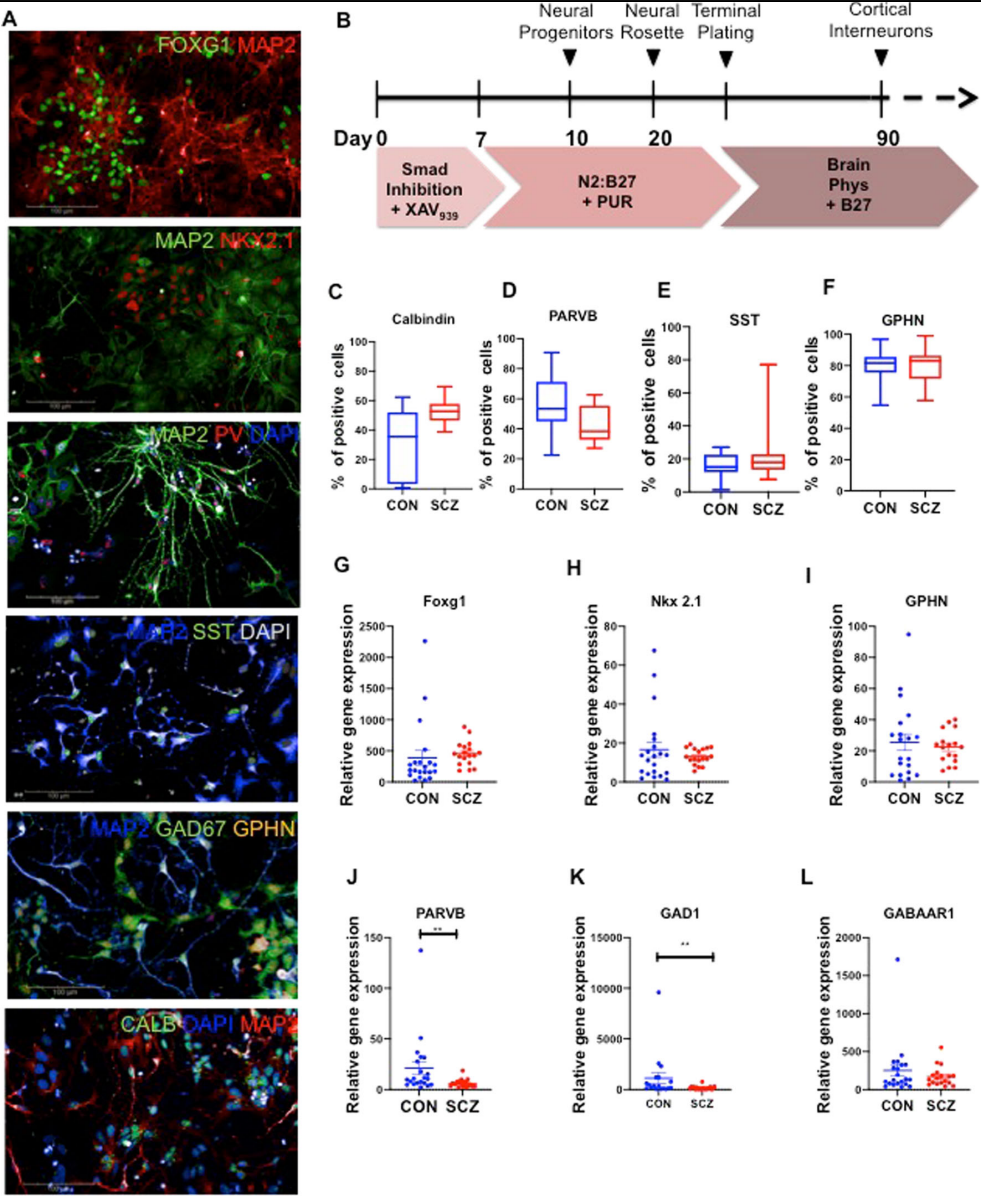
Differentiation and characterization of human iPSC-derived cortical interneurons. **a** Representative image of cortical interneurons at day 90 of differentiation with antibodies against FOXG1, Nkx2.1, parvalbumin (PV), somatostatin (SST), GAD67, gephyrin (GPHN), calbindin (CALB) and MAP2. Scale bar: 100 μm. **b** Schematic diagram of the differentiation protocol. **c**–**f** Quantification of cells expressing calbindin, parvalbumin, somatostatin and gephyrin in cortical interneurons in nine CON and seven SCZ lines. Ratios shown are calculated as number of marker-positive cells divided by total of number of nuclei. (**g**–**m**) Gene-expression data for FOXG1, Nkx2.1, GPHN, PARVB, GAD1, GABAAR1 and SST in seven CON and six SCZ lines, each with three replicates. Graphs show relative gene expression in cortical interneuron cultures compared to a control iPSC line assessed via quantitative PCR (qPCR). Values are mean±SEM. Mann–Whitney *U*-test was performed. The only significant differences between CON and SCZ groups were in PARVB (**p = 0.0019) and GAD1 (**p = 0.0064).

With approval from the Institutional Review Boards (IRB), we recruited age- and sex-matched SCZ and CON subjects to obtain fibroblasts and reprogramme them into iPSCs through induction with modified mRNA^17,18^. Supplementary Table 3 describes the iPSC lines used in this study, along with details of the subjects’ diagnoses, age, antipsychotic treatment history and medical co-morbidites. We validated reprogrammed iPSCs using standard protocols (Supplementary Fig. S1) and differentiated them to generate cortical interneurons using established protocols^19^. We confirmed cortical interneuron identity using immunocytochemistry and gene expression (Fig. 1). At day 90 of differentiation, the cultures were positive for neurons expressing FOXG1, Nkx2.1, parvalbumin (PARVB), somatostatin (SST), GAD67, gephyrin (GPHN) and calbindin (CALB). We quantified the these markers in all lines and ~80% of the cells expressed gephyrin, confirming their inhibitory nature (Fig. 1a, f). >40% of the cells were positive for calbindin, a calcium-binding protein found in inhibitory interneurons (Fig. 1a, c). Somatostatin was expressed in ~20% of neurons, while parvalbumin was expressed in 40–50% of the cells (Fig. 1a, d, e). Gene expression analysis with qPCR showed presence of FOXG1, Nkx2.1, GPHN, PARVB, GAD1, GABAAR1, and SST in all lines (Fig. 1g–m). While the fraction of cells positive for parvalbumin was similar in CON and SCZ (Fig. 1d), expression level of PARVB mRNA was significantly lower in SCZ compared to CON (Fig. 1j). A similar pattern has been reported in SCZ brains—density of parvalbumin expressing neurons was not different in SCZ but PARVB mRNA levels were significantly lower in the prefrontal cortex^15,20^. We found that GAD1 levels were significantly lower in SCZ interneurons (Fig. 1k), which recapitulates well-replicated postmortem findings of reduced levels of GAD1 expression in the prefrontal cortex in SCZ^21,22^.

### Effects of oxidative stress in cortical interneurons

**Fig. 2:**
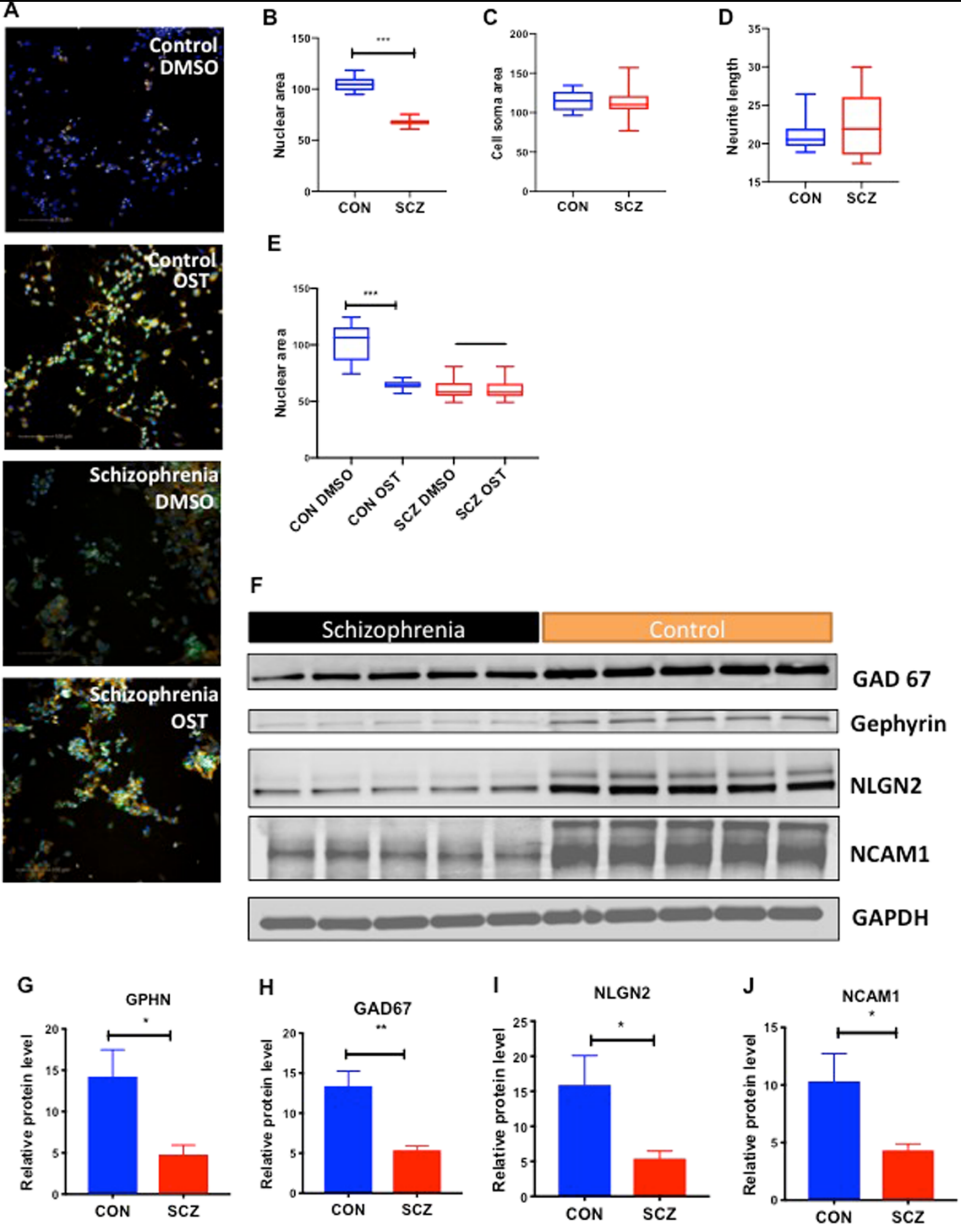
SCZ interneurons show disease-specific differences related to oxidative stress and have decreased expression of GABAergic and synaptic proteins. **a** Representative images of cortical interneurons with reactive oxygen species dye (ROS in green), super oxygen species dye (SO orange) and Hoechst 33342 nuclear stain (blue). Oxidative stress (OST) was induced using pyocyanin 0.1 mM. Scale bar: 100 μm. **b**–**d** Nuclear area (μm^2^), cell soma area (μm^2^), and average neurite length (μm) for interneuron cultures are shown. Unpaired Student’s *t*-test with Welch’s correction was performed. CON vs SCZ ***p < 0.0001 for nuclear area. **e** Effect of pyocyanin 0.1 mM on nuclear area. Unpaired Student’s *t*-test with Welch’s correction. CON: DMSO vs OST ***p < 0.0001. **f** Western blots of lysates from cortical interneurons of SCZ and CON subjects. **g**–**j** Quantification of protein levels normalized to GAPDH. Unpaired Student’s *t*-test with Welch’s correction. GPHN: CON vs SCZ *p = 0.0211; GAD67: CON vs SCZ **p = 0.0032; NLGN2: CON vs SCZ *p = 0.0380; NCAM1: CON vs SCZ *p = 0.0380.

We compared SCZ and CON cortical interneurons for cell soma area, nuclear area and neurite length (Fig. 2). The nuclear area in SCZ interneurons was significantly smaller when compared to CON (Fig. 2b) but cell soma area and neurite lengths did not vary between the groups (Fig. 2c, d). There is a large body of research indicating the presence of oxidative stress in SCZ, specifically in cortical interneurons^23–27^. Interneurons are highly susceptible to oxidative stress^28^ and oxidative stress affects the structure of the nuclear extracellular matrix and the nuclear area^29^. Our results suggested that SCZ interneurons have an innate oxidative stressed state at baseline. We examined the effect of oxidative stress with pyocyanin on cortical interneurons by measuring reactive oxygen species (ROS) and super oxygen species (SO). Pyocyanin (0.1 mM) increased ROS and SO species in both CON and SCZ cultures (Fig. 2a), which led to significant reduction in nuclear area in CON neurons but not in SCZ neurons (Fig. 2e). This suggests that SCZ interneurons have an innately stressed biology at baseline compared to CON interneurons and SCZ interneurons are restricted in their ability to modulate their nuclear area in response to oxidative stress.

### GAD67 and gephyrin levels are reduced in SCZ cortical interneurons

Postmortem studies in SCZ have shown significant alterations of key enzymes involved in synthesis of gamma-aminobutyric acid (GABA)^30^. It has not been clear whether these findings in postmortem brains were contributing to the disease biology of SCZ or whether they were downstream consequences of disease processes or treatment. iPSC-derived SCZ cortical interneurons showed significantly lower levels of GAD67 protein, an enzyme that decarboxylates glutamate to GABA (Fig. 2f, h), as well as reduced levels of GAD1 mRNA (Fig. 1k), consistent with postmortem SCZ findings^30,31^. These results indicate that the complex genetic background of SCZ predisposes interneurons to decreased expression of GAD67. SCZ cortical interneurons also expressed significantly lower levels of gephyrin, a postsynaptic protein specific to inhibitory neurons (Fig. 2f, g).

### Synaptic deficits in ex vivo co-cultures of SCZ cortical excitatory neurons and interneurons

**Fig. 3:**
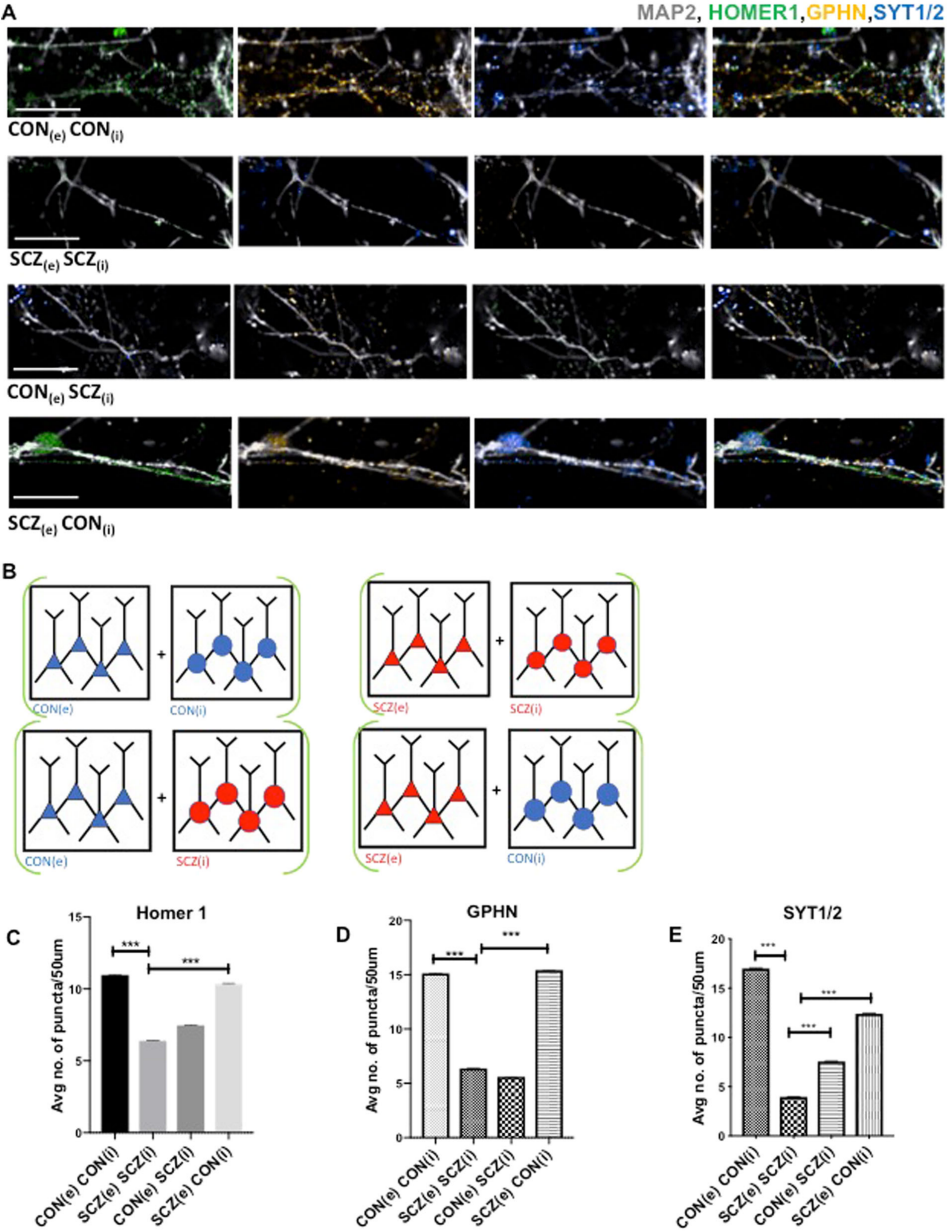
Co-cultures of cortical interneuron and excitatory neurons show decreased synaptic puncta density in SCZ. **a** Representative images of dendritic processes in co-cultures of excitatory and inhibitory neurons. Subscripts e and i denote excitatory and inhibitory neurons respectively. The following co-culture combinations are shown: CON_(e)_:CON_(i)_, SCZ_(e)_:SCZ_(i)_, CON_(e)_:SCZ_(i)_ and SCZ_(e)_:CON_(i)_. The first three columns show MAP2 (grey) staining with Homer1 (green), gephyrin (GPHN) (yellow), and Synaptotagmin1/2 (SYT1/2) (blue) respectively. The last column shows the cultures with all four markers included. Scale bar: 20 μm. **b** Schematic depiction of the four co-culture combinations studied. **c**–**e** Automated quantification of GPHN, Homer 1 and SYT1/2 puncta in dendrites from two CON and two SCZ lines (three replicates each for each line). Values are mean ± SEM. In all, >25,000 neurites analysed per condition. Unpaired Student’s *t*-test with Welch’s correction. Homer 1: CON_(e)_CON_(i)_ vs SCZ_(e)_SCZ_(i)_ ***p < 0.0001; SCZ_(e)_SCZ_(i)_ vs SCZ_(e)_CON_(i)_ ***p < 0.0001. GPHN: CON_(e)_CON_(i)_ vs SCZ_(e)_SCZ_(i)_ ***p < 0.0001; SCZ_(e)_SCZ_(i)_ vs SCZ_(e)_CON_(i)_ ***p < 0.0001. SYT1/2: CON_(e)_CON_(i)_ vs SCZ_(e)_SCZ_(i)_ ***p < 0.0001; SCZ_(e)_SCZ_(i)_ vs SCZ_(e)_CON_(i)_ ***p < 0.0001.

Since gephyrin is a neuronal assembly protein that anchors inhibitory neurotransmitter receptors to the postsynaptic cytoskeleton^32^, we examined whether there were differences in measures of synaptic connectivity in SCZ. Postmortem studies in SCZ show reduction in postsynaptic elements in the cortical but not in subcortical tissue^33^. We generated excitatory cortical neurons from the same iPSC lines using dual SMAD inhibition^34,35^ and co-cultured them with inhibitory cortical interneurons (Fig. 3). We quantified gephyrin (GPHN), Homer 1, and synaptotagmin1/2 (SYT1/2) in the co-cultures (Fig. 3a, b). SCZ co-cultures showed significantly lower density of GPHN, Homer 1, and SYT1/2 puncta when compared to CON co-cultures (Fig. 3c–e). We then “cross-cultured” SCZ and CON excitatory and inhibitory neurons to investigate how co-culturing SCZ interneurons with CON excitatory neurons, and vice versa, would affect synaptic puncta density (Fig. 3a, b). SCZ(e)-CON(i) co-cultures had synaptic puncta density similar to CON(e)-CON(i) while CON(e)-SCZ(i) densities were in the range seen with SCZ(e)-SCZ(i) co-cultures (Fig. 3c–e). These experiments showed that the decreased synaptic puncta density in SCZ co-cultures result from a deficiency inherent in SCZ interneurons.

### Decreased synaptic adhesion proteins in SCZ cortical interneurons

Changes in genes expressing synaptic adhesion proteins such as neural cell adhesion molecules and neuroligins have been implicated in SCZ^36^. In light of lower levels of GAD67 and gephyrin and reduced synaptic puncta density in SCZ interneurons, we examined whether synaptic adhesion and cytoskeletal proteins involved in synapse formation and maintenance were altered in SCZ interneurons. We found significant reduction in levels of Neural Cell Adhesion Molecule 1 (NCAM1) and Neuroligin 2 (NLGN2) in SCZ interneurons when compared to CON (Fig. 2f, i, j). NCAM1 plays an important role in molecular organization of the synaptic terminal and interacts with gephyrin to stabilize glycine and GABAA receptors at inhibitory synapse^36,37^. NLGN2 is expressed exclusively in inhibitory synapses, in contrast to NLGN1 and NLGN3^38^. These findings indicate that SCZ interneurons have innate deficiencies in the machinery for creating and maintaining the inhibitory synapse that could lead to decreased levels of synaptic connections and neuronal connectivity.

### NLGN2 loss-of-function in healthy neurons leads to reduced synaptic puncta density while NLGN2 overexpression rescues synaptic deficits in SCZ

**Fig. 4:**
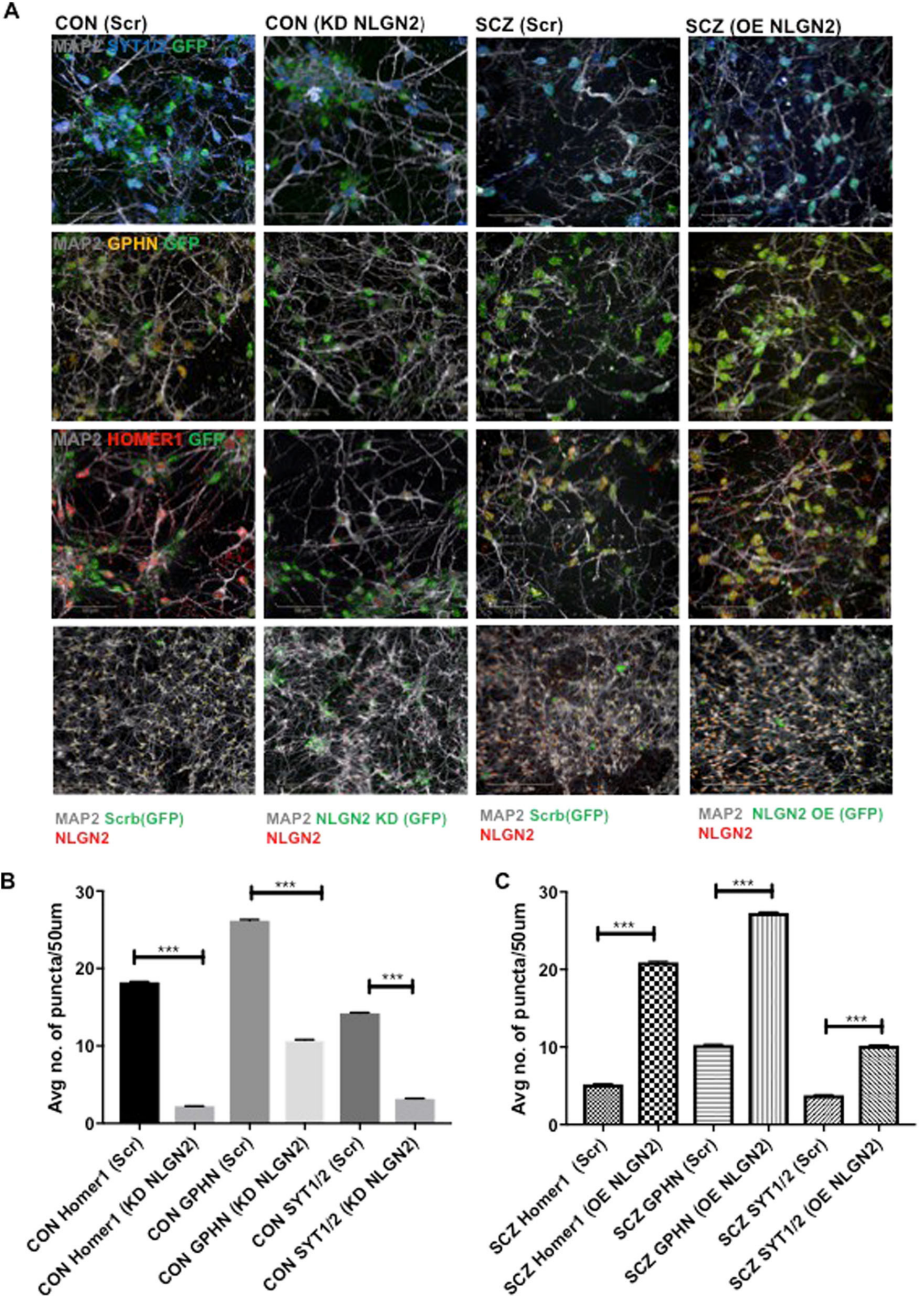
NLGN2 knockdown in healthy neurons recapitulates synaptic puncta deficits observed in schizophrenia neurons while NLGN2 overexpression in schizophrenia neurons restores synaptic puncta deficits. **a** Representative images of the co-cultures studied. CON_(e)_:CON_(i)_ co-cultures were transduced with NLGN2 GFP knockdown lentivirus or a scrambled control GFP lentivirus while SCZ_(e)_:SCZ_(i)_ co-cultures were transduced with NLGN2 overexpression (OE) lentivirus or scrambled control GFP lentivirus. Cultures are labelled with antibodies against GFP (green), MAP2 (grey), GPHN (yellow), Homer1 (red) and SYT1/2 (blue). Scale bar: 50 μm. **b** Quantification of Homer 1, GPHN and SYT1/2 puncta density in CON_(e)_:CON_(i)_ cultures with NLGN2 knockdown. Data collected from three replicates in each condition and values are shown as mean ± SEM. In all, >25,000 neurites analysed per condition. Unpaired Student’s *t*-test with Welch’s correction: CON Homer1(Scr) vs CON Homer1 (KD NLGN2) ***p < 0.0001; CON GPHN (Scr) vs CON GPHN (KD NLGN2) ***p < 0.0001; CON SYT1/2 (Scr) vs CON SYT1/2 (KD NLGN2) ***p < 0.0001. **c** Quantification of Homer 1, GPHN and SYT1/2 puncta density in the SCZ_(e)_:SCZ_(i)_ cultures in the setting of NLGN2 overexpression. Data collected from three replicates in each condition and values are shown as mean ± SEM. >25,000 neurites analysed per condition. Unpaired Student’s *t*-test with Welch’s correction: SCZ Homer1(Scr) vs SCZ Homer1 (OE NLGN2), ***p < 0.0001; SCZ GPHN (Scr) vs SCZ GPHN (OE NLGN2) ***p < 0.0001; SCZ SYT1/2 (Scr) vs SCZ SYT1/2 (OE NLGN2) ***p < 0.0001.

NLGN2 has a pivotal role in the inhibitory synaptic structure^38^. Since decreased NLGN2 accompanied reduced synaptic puncta density in SCZ interneurons, we sought to to determine whether differential expression of NLGN2 was mediating the reduction in synaptic puncta density. We carried out loss-of-function studies of NLGN2 in CON cultures, utilizing shRNA lentivirus against NLGN2 (Fig. 4). NLGN2 knockdown in CON cultures resulted in significant reduction of Homer 1, GPHN and SYT1/2 puncta (Fig. 4a, b), mirroring results observed in SCZ interneurons (Fig. 2f, i). We then carried out the converse experiment by overexpressing NLGN2 in SCZ neurons, which led to a significant increase in Homer 1, GPHN and SYT1/2 puncta (Fig. 4a, c). These results show that NLGN2, which is decreased in SCZ interneurons, mediates synaptic puncta density reduction in SCZ neurons.

### SCZ interneurons show reduced spontaneous activity

**Fig. 5:**
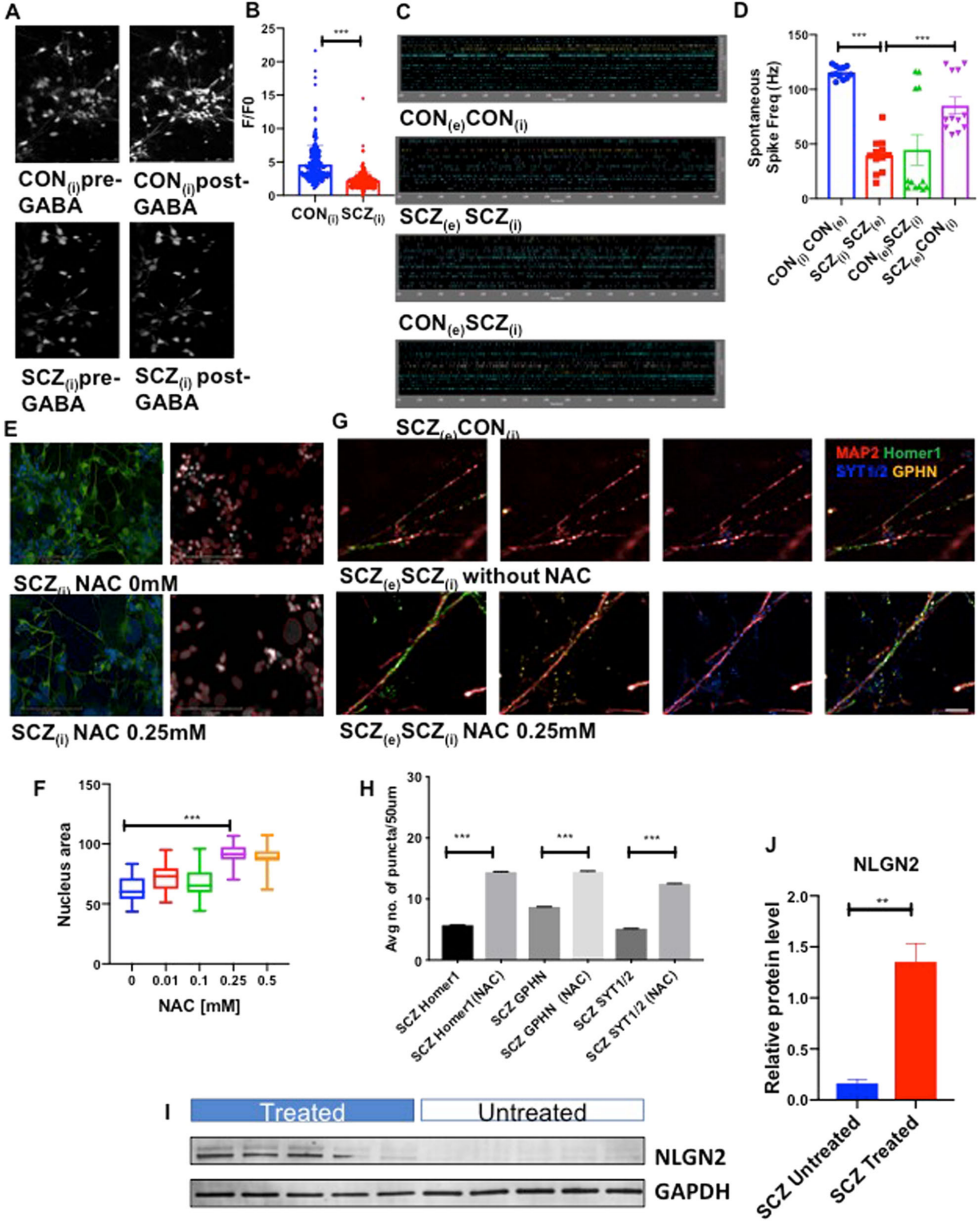
Functional characterization of interneurons show disease-specific deficits in SCZ while NAC increases NLGN2 expression and rescues synaptic deficits in schizophrenia interneurons. **a** Time-lapse images of Ca^2+^ dye (Fluo-4AM) before and after exposure to GABA in CON and SCZ interneurons. Scale bar: 50 μm. **b** Fluorescence intensity of Fluo-4AM after exposure to GABA [F] divided by fluorescence intensity before addition of GABA [F(0)]. Data collected for six CON and six SCZ lines (three replicates each). Values are mean±SEM. Mann–Whitney *U*-test was performed: CON_(i)_ vs SCZ_(i)_ ***p < 0.0001. **c** Representative images of raster plots from microelectrode array experiments measuring spontaneous electrical activity in co-cultures of excitatory and inhibitory neurons. **d** Spontaneous spike frequency (Hz) data collected for two CON and two SCZ lines (three technical replicates for each line). Values are mean±SEM. Unpaired Student’s *t*-test with Welch’s correction: CON_(e)_CON_(i)_ vs SCZ_(e)_SCZ_(i)_ ***p < 0.0001; SCZ_(e)_SCZ_(i)_ vs SCZ_(e)_CON_(i)_ ***p < 0.0001; SCZ_(e)_SCZ_(i)_ vs SCZ_(e)_CON_(i)_ ***p < 0.0001. **e** Representative images of SCZ interneurons treated with NAC and stained with neuronal marker Tuj1 (green) and nuclear marker DAPI (blue) along with skeletized images for analyses. Scale bar: 100 μm. (**f**) Nuclear area (μm^2^) of SCZ interneurons with exposure to different doses of NAC. Data collected for eight SCZ lines (three replicates each) and values are mean ± SEM. One-way ANOVA with Dunnett’s multiple comparison test: 0 mM NAC vs 0.25 mM NAC ***p < 0.0001. **g** Synaptic puncta in SCZ co-cultures of excitatory and inhibitory neurons, labelled with antibodies against Homer1 (green), GPHN (yellow), MAP2 (red) and SYT1/2 (blue), in the presence and absence of 0.25 mM NAC. **h** Quantification of Homer 1, GPHN and SYT1/2 puncta density in co-cultures of excitatory and inhibitory neurons from two SCZ lines (three replicates each), in the presence and absence of 0.25 mM NAC. >25,000 neurites analysed per condition. Values are mean ± SEM. Unpaired Student’s *t*-test with Welch’s correction: SCZ Homer 1 vs SCZ Homer 1 (NAC) ***p < 0.0001; SCZ GPHN vs SCZ GPHN (NAC) ***p < 0.0001; SCZ SYT1/2 vs SCZ SYT1/2 (NAC) ***p < 0.0001. **i** Western blots of NLGN2 in interneurons from five SCZ lines, in the presence and absence of 0.25 mM NAC. **j** Quantification of protein levels from **I**. Values are mean ± SEM. Unpaired Student’s *t*-test with Welch’s correction: **p = 0.0023.

In light of the reduced synaptic density in SCZ interneurons, we sought to characterize functional activity in SCZ and CON neuronal cultures. We measured Ca^2+^ oscillations under baseline conditions and in setting of GABA exposure (Fig. 5). CON interneurons had a much more robust response to GABA compared to the SCZ interneurons (Fig. 5a, b). We also used a microelectrode array (MEA) to record extracellular action potential waveforms and quantify the frequency of spontaneous action potential spikes in a real-time and in a label-free manner. To verify that MEA was accurately recording neuronal activity, we confirmed that 1 μM tetrodotoxin (TTX) abolished neuronal electrical activity (Supplementary Figure S2F, G). In MEA experiments, SCZ co-cultures had significantly lower frequency of spontaneous activity compared to CON neurons (Fig. 5c, d). We then cross-cultured SCZ and CON interneurons and excitatory neurons and recorded extracellular action potential waveforms. Co-culturing CON interneurons with SCZ excitatory neurons resulted in a frequency of spontaneous activity similar to that observed with the CON co-cultures while co-culturing SCZ interneurons with CON excitatory neurons resulted in a frequency of spontaneous activity similar to that observed with the SCZ co-cultures. These results are consistent with results of the synaptic puncta co-culture studies (Fig. 3) and indicate that differences in the electrical activity between the SCZ and CON neuronal cultures are attributable to deficits in SCZ cortical interneurons.

### N-acetyl cysteine rescues morphological and synaptic puncta deficits in SCZ interneurons

*N*-acetyl cysteine (NAC) is an antioxidant with efficacy in treating symptoms relevant to schizophrenia, both in preclinical models and in clinical studies^39–43^. Since our studies indicated that SCZ interneurons have an innately stressed biology at baseline, reflected in the reduced nuclear area (Fig. 2b), we examined the effect of NAC on the nuclear area in SCZ. We carried out a dose-response experiment and found that exposure to 0.25 mM NAC for 24 h resulted in significant increase in nuclear area of SCZ interneurons (Fig. 5f). Exposure to 0.25 mM NAC in SCZ co-cultures also led to significant increase in the density of Homer 1, GPHN and SYT1/2 puncta (Fig. 5g, h). This suggests that decreasing oxidative stress with NAC in SCZ cultures leads to increased expression of synaptic markers. Since we had earlier found that differences in NLGN2 expression modulated levels of synaptic puncta density in the co-cultures, we examined whether NAC affected NLGN2 levels in mediating the increase in synaptic puncta density. SCZ interneurons exposed to 0.25 mM NAC showed a significant increase in NLGN2 levels (Fig. 5i, j). Collectively, these results showed that ameliorating oxidative stress with NAC in SCZ leads to increased NLGN2 expression in interneurons and restores synaptic deficits in SCZ neuronal cultures.

## Discussion

Advances in our understanding of the disease biology of SCZ have been hindered by the difficulty in generating cell types relevant to the disease and in identifying disease-specific abnormalities in cells from patients with complex psychiatric disorders^44^. Cellular reprogramming methods enable generation of human iPSCs, which can be differentiated to neuronal subtypes implicated in the biology of psychiatric disorders^45–51^. Recent methodological advances in human iPSC differentiation enable generation of cortical interneurons implicated in SCZ disease biology^19,52^. We differentiated iPSCs to cortical interneurons from 18 individual subjects to examine disease-specific differences in specific neuronal subtypes.

We show here that SCZ interneurons express lower levels of GAD67 and gephyrin and have a reduced density of synaptic puncta in co-cultures with excitatory neurons. In doing so, we show that a well-replicated post-mortem finding in SCZ, that of lower levels of GAD67, can be recapitulated in neuronal cultures generated from iPSCs of SCZ subjects. By cross-culturing inhibitory and excitatory neurons, we show that the decreased synaptic puncta in SCZ result from deficiencies in SCZ cortical interneurons. We further show that lower levels of NLGN2 accompany the decreased synaptic puncta density in SCZ. With knockdown and overexpression experiments, we show that NLGN2 expression mediates the reduction in synaptic puncta density in SCZ. Ca^2+^ imaging and MEA studies revealed that the functional decifits in SCZ neurons arise from deficits inherent in the interneurons. Taken together, our findings implicate deficiencies in the synaptic machinery in cortical interneurons as a critical regulator of excitatory and inhibitory (E-I) activity imbalance in SCZ.

We also discovered a heretofore-unknown connection between oxidative stress and synaptic connections in SCZ mediated by NLGN2. SCZ interneurons, which have a smaller nuclear area indicative of an innate oxidative stressed state, do not modulate their nuclear area in response to oxidative stress, unlike CON interneurons. There is a large body of literature on the role of oxidative stress in schizophrenia^23–28^ and the potential of NAC in treating symptoms relevant to schizophrenia^39–43^. We found that NAC increased nuclear area in SCZ interneurons in a dose-dependent manner to values in observed in CON interneurons. Futhermore, NAC increased NLGN2 levels in SCZ interneurons and led to increased synaptic puncta density. While previous literature had reported deficits in oxidative stress in SCZ, we show for the first time that ameliorating oxidative stress with NAC leads to significant effects on synaptic biology in SCZ.

There are notes of caution in generalizing results from cellular models of idiopathic complex brain disorders such as SCZ. There are continuing debates about whether SCZ constitutes one major disease entity that has the same underlying biology or whether it is a syndrome that comprises of multiple disorders with different causes^53^. It is possible that there may be different biological processes involved in the development of the disease in different subsets of patients. Nevertheless, the clinical presentation of SCZ is rather stereotyped and most cases of this condition may share substantial mechanisms at the genomic, cellular and circuitry levels. The GABAergic hypothesis tested here was based on postmortem findings from patients who had been subjected to the physiological stress of psychotic episodes as well as that of the effects of medications over the years^20,21,30,31^. The iPSC reprogramming process results in erasure of much of the epigenetics and hence, it only enables us to capture the risks and features accorded by the underlying complex genetics and not from epigenetic differences^54^. Since our ex vivo findings recapitulate the postmortem results, this suggests that the postmortem findings of GABAergic deficits in SCZ reflect underlying genetic differences. Pursuit of studies like the ones reported here might lead to a better characterization of those underlying mechanisms and the identification of targets for new therapeutic approaches.

## Supplementary information

Supplemental Material
