# Supplementary material for: Synaptic deficits in iPSC-derived cortical interneurons in schizophrenia are mediated by NLGN2 and rescued by N-acetylcysteine
Source: Transl Psychiatry. 2019 Nov 28;9:321. doi: 10.1038/s41398-019-0660-x (PMC6882825; doi:10.1038/s41398-019-0660-x)
Supplement: Supplementary file 1 — Supplemental Material [file 41398_2019_660_MOESM1_ESM.pdf]

Supplementary Figure 1

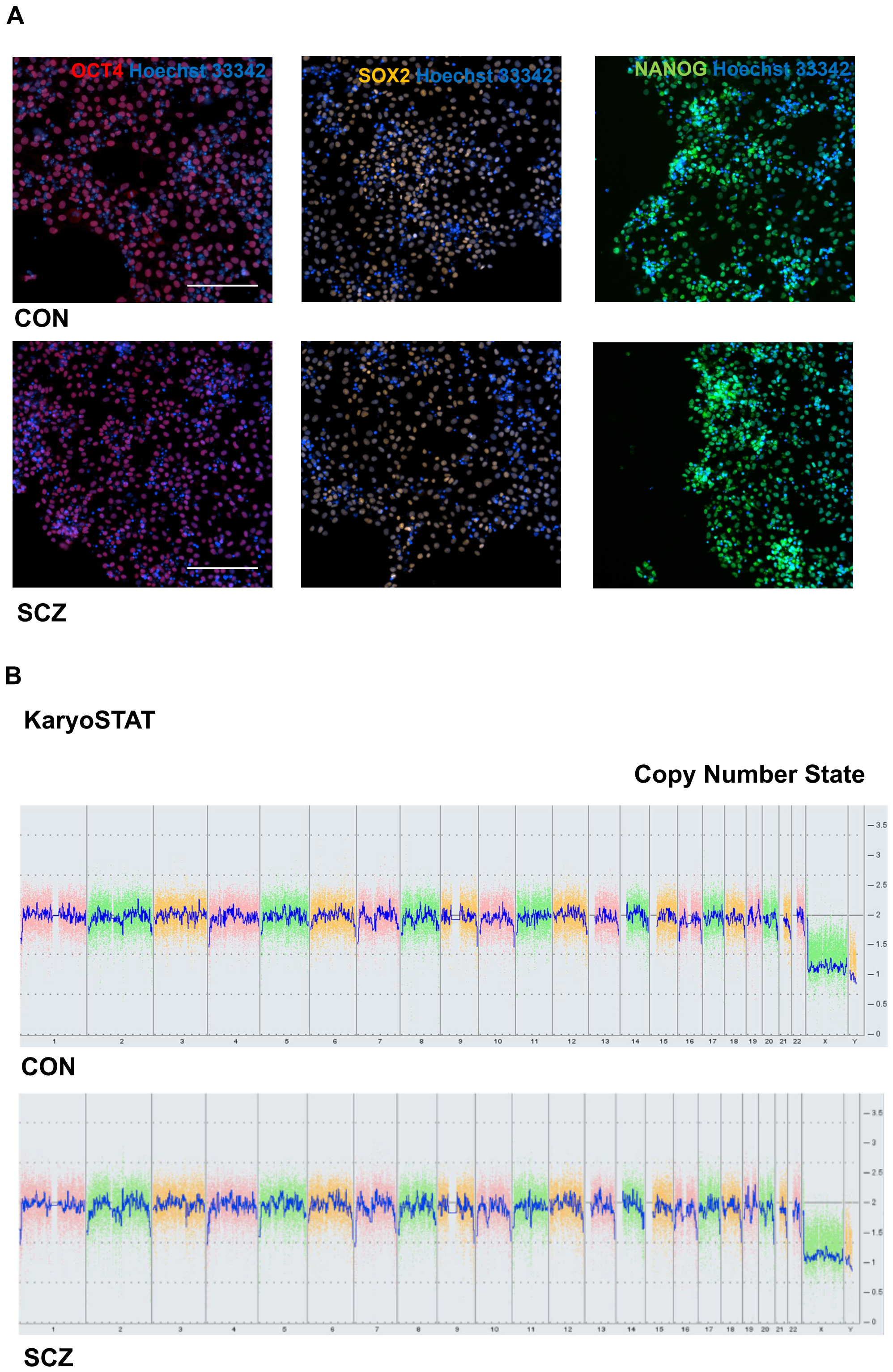

(A) Representative images of immunocytochemistry of CON and SCZ iPSC lines showing expression of pluripotency markers Nanog (green), Oct4 (red), Sox2 (yellow), and Hoechst 33342 (blue). Scale bar: 50μm. This characterization was done for all 18 iPSC lines. (B) Karyotyping with KaryoSTAT assay. Representative images of the whole-genome view of one CON line and one SCZ line are shown. The whole genome view displays all somatic and sex chromosomes in one frame with high level copy number. The smooth signal plot (right y-axis) is the smoothing of the log2 ratios which depict the signal intensities of probes on the microarray. A value of 2 represents a normal copy number state (CN = 2). A value of 3 represents chromosomal gain (CN = 3). A value of 1 represents a chromosomal loss (CN = 1). Pink, green and yellow colors indicate raw signals for each individual chromosome probe, while blue signal represents normalized probe signal which is used to identify copy number and aberrations (if any). No chromosomal aberrations were found when comparing against the reference dataset. Eight of the CON lines and eight of SCZ lines showed no chromosomal aberrations, however one CON line showed a gain in chromosome 2, and one SCZ line showed a gain in chromosome 12. These were excluded from analysis.

Supplementary Figure 2

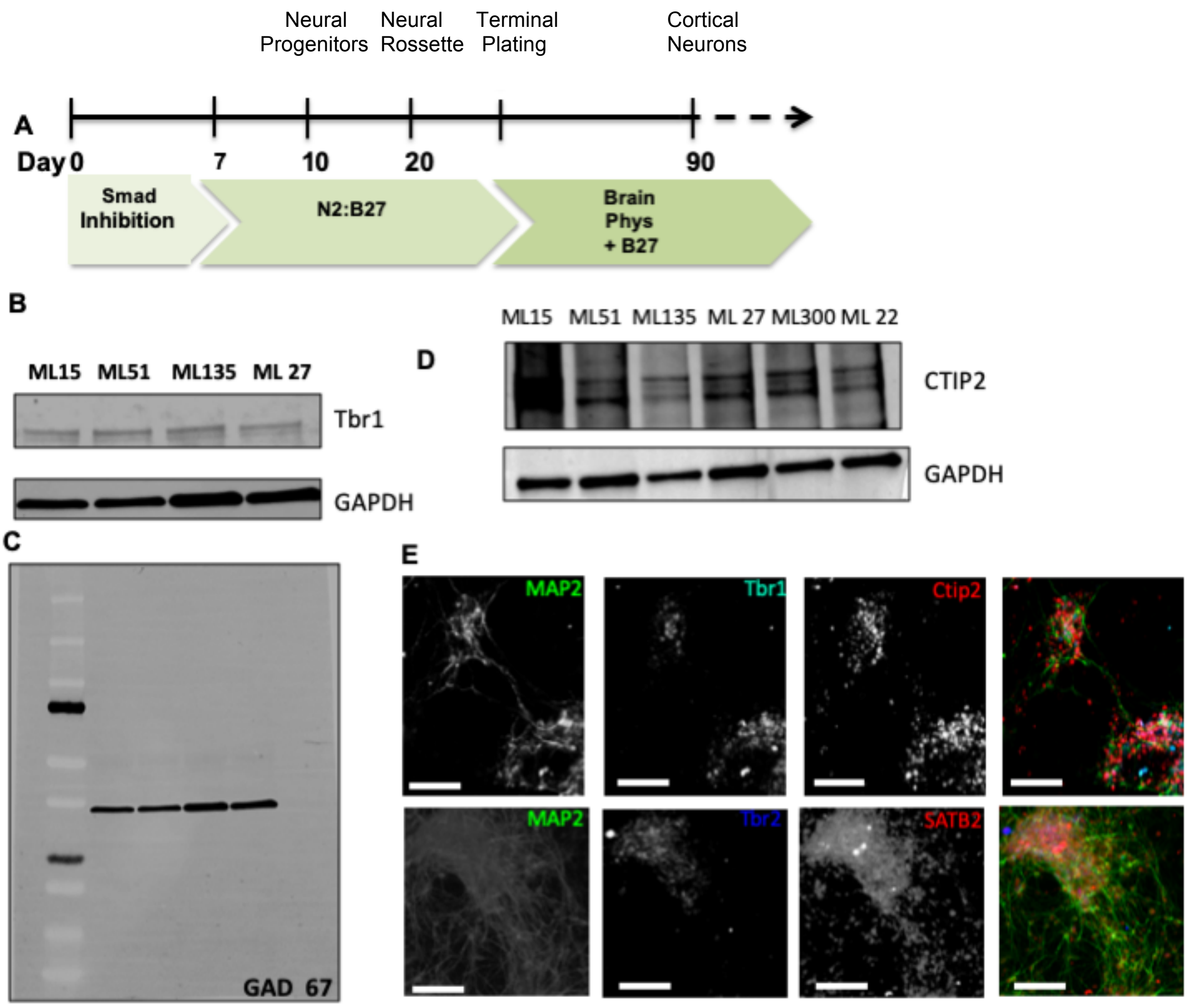

(A) Schematic diagram of differentiation protocol used to generate cortical excitatory neurons from iPSCs. (B-D) Western blots of cortical excitatory neurons at day 90 of differentiation, showing expression of cortical markers TBR1 and CTIP2 and no GAD67 expression. (E) Immunocytochemistry of representative cortical excitatory neurons with antibodies against MAP2 (green), TBR1 (cyan), TBR2 (blue), CTIP2 (red) and SATB2 (red). Scale bar = 100μm.

Supplementary Figure 3

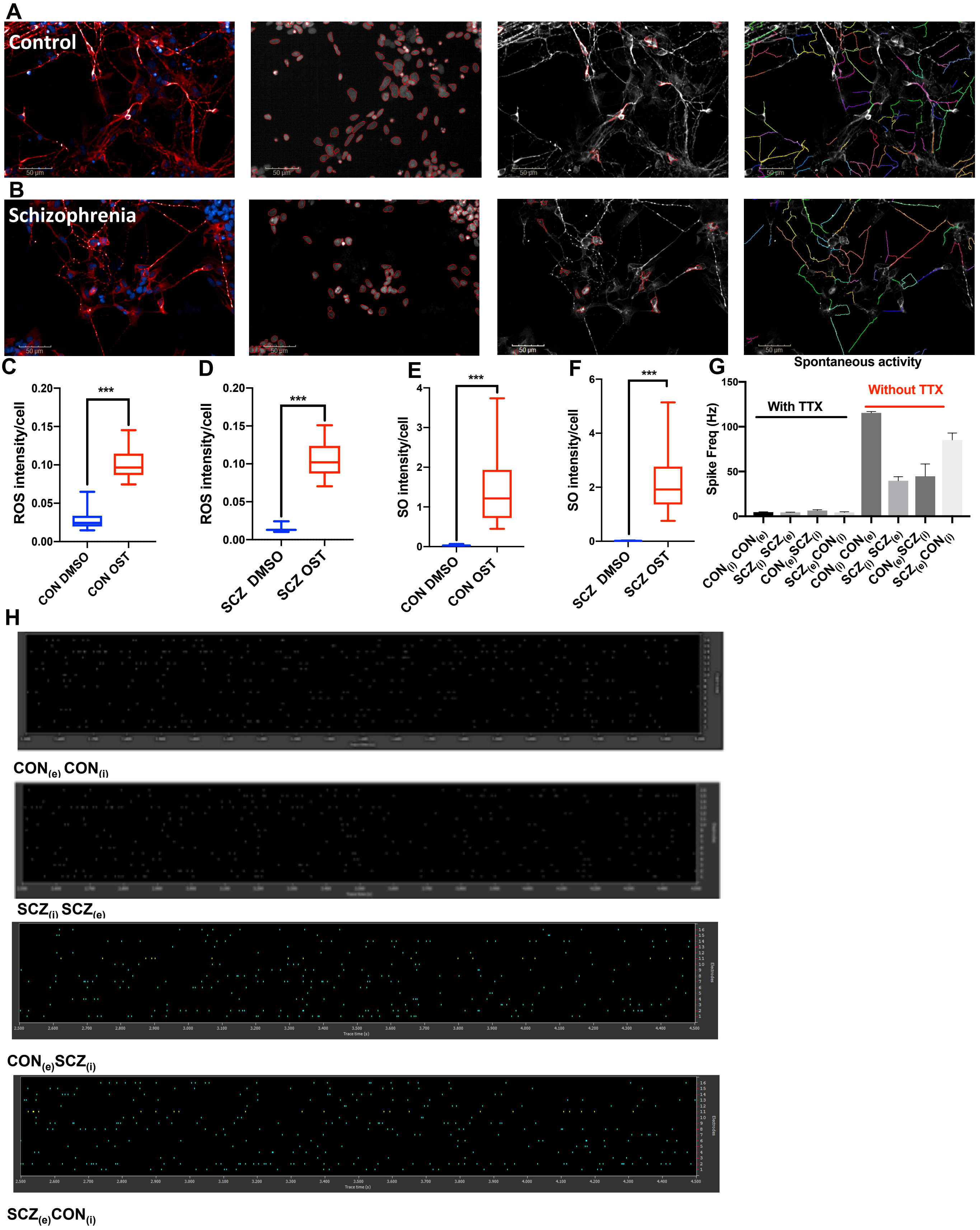

(A-B) Representative images of cortical interneurons stained with MAP2 (red) and Hoechst 33342 nuclear stain (blue). Skeletization of neurites for analysis is shown. Scale: 50μm. (C-F) Fluorescence intensity of ROS normalized to total nuclei for eight independent lines each for CON, SCZ cortical interneurons, each with 3 replicates. Unpaired student t-test with Welch's correction. CON: DMSO vs OST ***p<0.0001, SCZ: DMSO vs OST ***p<0.0001. (I-J) Fluorescence intensity of SO normalized to total nuclei. Unpaired student t-test with Welch's correction. CON: DMSO vs SO ***p<0.0001, SCZ: DMSO vs SO ***p<0.0001. G. Quantification of spike frequency with exposure to 1μm TTX. H. Raster plots of spontaneous activity recording for 60 seconds of CON and SCZ co-cultures.

Supplementary Table 1: Primary antibodies.

Primary antibody	Type	Dilution	Source	Catalogue #
**Calbindin**	Rabbit polyclonal	1/500	abcam	ab49899
**Ctip2 [25B6]**	Rat monoclonal	1/200	abcam	ab18465
**CUTL1 (521-620)**	Mouse monoclonal	1/300	Abnova	H00001523-M01
**EphA3/A4/A5 (D2c11)**	Rabbit monoclonal	1/1000 (WBs)	Cell Signaling Technology	8793S
**GAD 1/GAD 67 #1**	Affinity-purif. Rabbit	1/200 ICC<br>1/1000 WBs	Synaptic Systems	198 003
**GAPDH**	Mouse monoclonal	1/5000 WBs	proteintech	60004-l-lg
**Gephyrin (cl. mAb7)**	Mouse monoclonal, purif. lgG	1/200 ICC<br>1/1000 WBs	Synaptic Systems	147 011
**GFAP**	Chicken polyclonal	1/1000	abcam	ab4674
**Homer 1**	Chicken polyclonal, purif. IgY	1/200	Synaptic Systems	160 006
**MAP2**	Guinea pig polyclonal antiserum	1/1000	Synaptic Systems	188 004
**NCAM (CD56) (123C3)**	Mouse monoclonal	1/1000	Cell Signaling Technology	3576S
**Neuroligin 2**	Rabbit polyclonal, affinity purified	1/1000	Synaptic Systems	129 203
**PAX6 (Clone Poly19013)**	Rabbit polyclonal	1/500	BioLegend	901301
**PV**	Rabbit polyclonal		1/200	abcam
**SATB2 [SATBA4B10]**	Mouse monoclonal	1/200	abcam	ab11427
**Somatostatin (Clone YC7)**	Rat monoclonal	1/200	EMD Millipore Corp.	ab51502
**TBR1**	Rabbit polyclonal	1/200	abcam	MAB354
**Tubulin β 3 (TUBB3)**	Mouse monoclonal	1/1000	BioLegend	ab31940

Supplementary Table 2: List of Primers used.

Primer	Forward Sequence (5'-3')	Reverse sequence (5'-3')
**PARVB**	CTGAAGACGTGGTTAACTTG	CGTTCTTGTACTTGGTGAAC
**GABRA1**	ACTGTCTTTGGAGTAACAAC	CCATGCATAACCTCTCTTAG
**GABRB2**	CTATGGATACACAACTGATGAC	CTACAATAGAGAACTGTGGAAG
**GAD1**	CAATACCAACATGCCATCAG	ATTTAGGAGGAAGACCCTTG
**GAPDH**	ACAGTTGCCATGTAGACC	TTGAGCACAGGGTACTTTA
**RPL32**	GTGCAACAAATCTTACTGTG	CTGCCTACTCATTTTCTTCAC
**GPHN**	ACAGGTAATCAAATGAGCAG	TATGTGGAACATGCATCAAAG
**FOXG1**	TTGGGTTCTAGAAAATGCAC	TACACAACACAAACTGAAGG
**TTF1**	AAGGTCAGCCTTATTGAAAG	CGATGATCTCTGGAAAAGTC

Supplementary Table 3: Demographic and clinical data of subjects whose iPSC lines were used in the study.

Sample ID	Diagnosis	Gender	Age	Race	Age of onset	Antipsychotic history	Medical comorbidities
ML-15	Control	Male	37	Caucasian	-		
ML-22	Control	Male	24	Caucasian	-		
ML-27	Control	Male	24	Caucasian	-		
ML-33	SCZ	Male	32	Caucasian	22	clozapine, risperidal, ziprasidone,	None
ML-37	SCZ	Female	57	Caucasian	18	clozapine	hyperlipidemia
ML-40	SCZ	Female	46	Caucasian	13	clozapine, haloperidol	hypertension, hypothyroidism
ML-51	Control	Male	31	Caucasian	-		
ML-56	Control	Female	52	Caucasian	-		
ML-67	SCZ	Male	50	Caucasian	17	clozapine	
ML-123	SCZ	Male	32	Caucasian	-		
ML-135	Control	Male	49	Caucasian	-		
ML-141	SCZ	Male	40	Caucasian	24	aripiprazole	None
ML-164	SCZ	Female	67	Caucasian	unknown	perphenazine	unknown
ML-233	SCZ	Female	39	Caucasian	unknown	haloperidol	hypothyroidism
ML-250	SCZ	Male	23	Caucasian	unknown	aripiprazole	unknown
ML-292	control	Male	43	Caucasian	-		
ML-300	control	Female	32	Caucasian	-		
ML-364	control	Female	48	Caucasian	-		
